# Fish Out of Water: Junior doctors’ experiences of psychiatry.

**DOI:** 10.1192/j.eurpsy.2023.2386

**Published:** 2023-07-19

**Authors:** R. Holdsworth, H. Browne, R. Ilsted, J. Beezhold

**Affiliations:** 1 Great Yarmouth Acute Services, Northgate Hospital, Norfolk and Suffolk NHS Foundation Trust, Great Yarmouth, United Kingdom

## Abstract

**Introduction:**

This poster will explore the experiences of three junior doctors during their inpatient psychiatry placements. These doctors are Foundation Year 1, Foundation Year 2, and Foundation Year 3 doctors - i.e. not psychiatry trainees.

**Objectives:**

Due to the nature of the UK Foundation Programme, many FY doctors will not have chosen to work in psychiatry and will have been given the rotation as part of a package of jobs. Studies have shown that the risk of burnout is higher when a doctor working in psychiatry did not identify it as their top career choice (Jovanović, N. *et al.* (2016) *European Psychiatry*, 32, pp. 34–41). Lack of supervision is also a risk factor for burnout (Jovanović, N. *et al.* (2016) *European Psychiatry*, 32, pp. 34–41).

A phenomenological study(Beattie, S. *et al.* (2017) *BMJ Open*, 7(9)) demonstrated that job satisfaction and morale amongst junior doctors in psychiatry can be positively influenced by a sense of connectedness, clear role definition, structure and appropriate responsibility. Additionally, junior doctors’ experience of psychiatry, positive or negative, can influence their future career plans (Stott, J., Haywood, J. and Crampton, P. (2021) 43(10), pp. 1196–1202); this has important implications for recruitment into the specialty.

**Methods:**

Three junior doctors were interviewed. These consisted of two Foundation Year doctors, and one doctor who has completed FY2 but is working as a locum and not currently in training. They were asked about memorable experiences during their psychiatry placements. Additionally, they were asked about their emotions regarding work at the very start of their placements and towards the end.

**Results:**

The junior doctors that participated in interviews for the poster initially found the psychiatric inpatient setting challenging and overwhelming. Some of the challenges focused on the occasionally violent and risky nature of the ward and adjusting to that environment. However, all three doctors were pleasantly surprised by the way they adapted to the ward, the supportive nature of the team, and the rewarding experience of seeing very unwell patients get better.

**Conclusions:**

Overall the interviews demonstrated that there is a significant emotional impact on junior doctors working in psychiatry, particularly when it is their first experience of in-patient psychiatry. However, this emotional demand can be mitigated by a supportive multi-disciplinary team and good quality supervision.

**Disclosure of Interest:**

None Declared

